# Comparing the diagnostic efficacy of [^18^F]FDG PET/CT and [^18^F]FDG PET/MRI in breast cancer recurrence: a systematic review and meta-analysis

**DOI:** 10.3389/fmed.2025.1602415

**Published:** 2025-08-01

**Authors:** Shiqing Liu, Yu Xie, Wei Ding, Xiaopeng Ma, Zhilin Li, Lei Zhang, Ya Zhang, Conghui Ai

**Affiliations:** ^1^Department of Radiology, The Third Affiliated Hospital of Kunming Medical University (Yunnan Cancer Hospital, Yunnan Cancer Center), Kunming, Yunnan, China; ^2^The First College of Clinical Medicine, Gansu University of Chinese Medicine, Lanzhou, Gansu, China; ^3^920th Hospital of Joint Logistics Support Force, Kunming, Yunnan, China; ^4^The Second Hospital of Hebei Medical University, Shijiazhuang, Hebei, China; ^5^Department of Gynecology, The Third Affiliated Hospital of Kunming Medical University (Yunnan Cancer Hospital, Yunnan Cancer Center), Kunming, Yunnan, China

**Keywords:** breast cancer, [^18^F]FDG PET/CT, [^18^F]FDG PET/MRI, recurrence, meta-analysis

## Abstract

**Purpose:**

This meta-analysis evaluates and compares the diagnostic accuracy of [^18^F]FDG PET/CT and [^18^F]FDG PET/MRI in detecting breast cancer recurrence.

**Methods:**

A search was conducted across PubMed, Web of Science, and Embase databases up to June 10, 2025, to identify studies evaluating the diagnostic performance of [^18^F]FDG PET/CT and/or [^18^F]FDG PET/MRI in breast cancer recurrence. Sensitivity and specificity were calculated using the DerSimonian and Laird method with Freeman-Tukey double arcsine transformation. The Quality Assessment for Studies of Diagnostic Accuracy-2 (QUADAS-2) guidelines were employed to perform the quality evaluation.

**Results:**

Seventeen studies involving 1,450 patients were included. At the lesion level, the sensitivity of [^18^F]FDG PET/CT was 0.97 (95% CI: 0.91–1.00), with a specificity of 0.79 (95% CI: 0.58–0.94). [^18^F]FDG PET/MRI showed a sensitivity of 0.95 (95% CI: 0.91–0.99) and specificity of 0.87 (95% CI:0.75–0.95). Both modalities demonstrated similar sensitivity (*p* = 0.71) and specificity (*p* = 0.66). At the patient level, the sensitivity of [^18^F]FDG PET/CT was 0.93 (95% CI: 0.88–0.96), with a specificity of 0.87 (95% CI: 0.80–0.93). [^18^F]FDG PET/MRI showed a sensitivity of 0.99 (95% CI: 0.94–1.00) and specificity of 0.98 (95% CI, 0.90–1.00). Both modalities demonstrated similar sensitivity (*p* = 0.07) and specificity (*p* = 0.06).

**Conclusion:**

[^18^F]FDG PET/CT and [^18^F]FDG PET/MRI exhibit comparable sensitivity and specificity in detecting breast cancer recurrence at both the lesion and patient levels. However, high heterogeneity warrants further head-to-head studies to strengthen the evidence and provide more comprehensive insights.

## 1 Introduction

Among the top causes of female mortality, breast cancer remains a major global health concern (1, 2). According to projections by the American Cancer Society, approximately 313,510 individuals in the United States are expected to be diagnosed with breast cancer in 2024, while an estimated 42,780 are anticipated to succumb to the disease (3). In the 10 years following initial treatment, at least 35% of breast cancer patients experience local or distant recurrence (4). Breast cancer frequently recurs locally and metastasizes distantly, leading to poor prognosis and high mortality. Therefore, it is essential for patients to get a timely diagnosis of local recurrences and distant metastases.

Breast cancer recurrence is generally evaluated with a physical exam, as well as a mammogram, computed tomography (CT), magnetic resonance imaging (MRI), and bone scintigram. Despite detecting masses and lymph node abnormalities, physical examinations lack sensitivity to detect small metastases and early recurrences, complicating accurate staging (5). Both CT and MRI are susceptible to false positives due to postoperative fibrosis and scarring, and their resolution may be insufficient for small lesions (6). Although bone scans are highly sensitive for detecting bone metastases but they lack specificity, which can result in misdiagnoses due to non-cancerous conditions (7). These methods frequently struggle to distinguish between postoperative changes and true pathology, especially in early or small recurrences.

To address these limitations, recent advancements in multimodal and molecular imaging have introduced new approaches for detecting breast cancer recurrence and metastasis. [^18^F] Fluorodeoxyglucose positron emission tomography/CT ([^18^F]FDG PET/CT) and [^18^F]FDG PET/MRI are rapidly advancing molecular imaging technologies widely used for detecting and localizing breast cancer recurrence. [^18^F]FDG is a radiotracer that determines cellular metabolism by exploiting *in vivo* metabolic properties, which enables it to detect tumors and abnormal tissues (8). [^18^F]FDG PET/CT combines positron emission tomography and computed tomography, offering precise metabolic data and high-resolution anatomical images, crucial for identifying and analyzing recurrent lesions (9). [^18^F]FDG PET/MRI integrates positron emission tomography with magnetic resonance imaging, providing both metabolic and functional information, potentially advantageous for studying the biological traits of recurrent lesions (10). In addition, PET/MR excels in soft tissue contrast, making it more sensitive to vascular and soft tissue diseases (11). However, the relative diagnostic performance of these technologies remain debated.

In this study, articles with [^18^F]FDG PET/CT and [^18^F]FDG PET/MRI results have been evaluated and compared to detect recurrent breast cancer. A direct comparison in this context has not been thoroughly explored, making this study an important contribution to the understanding of imaging techniques in breast cancer recurrence evaluation.

## 2 Methods

This meta-analysis followed the Preferred Reporting Items for Systematic Reviews and Meta-analyses for Diagnostic Test Accuracy (PRISMA-DTA) guidelines for diagnostic test accuracy (12). PROSPERO network (ID CRD42024556069) prospectively registered the protocol.

### 2.1 Search strategy

A comprehensive search was performed across PubMed, Web of Science, and Embase databases to identify relevant articles, covering studies published up to June 10, 2025. The search queries included the following key terms: “Breast Neoplasms,” “Positron-Emission Tomography,” and “Recurrence.” Supplementary Table S1 provides details on the search strategy. To locate any further relevant studies, we manually searched the references of the retrieved articles.

### 2.2 Inclusion and exclusion criteria

In accordance with the PICOS (participants, intervention, control, outcomes, study design) framework, we included the following elements in our study: (1) Participants (P): Patients with suspected recurrence in breast cancer; (2) Intervention (I): Studies employing [^18^F]FDG PET/CT; (3) Comparisons (C): Studies comparing with [^18^F]FDG PET/MRI; (4) Outcomes (O): Studies reporting on sensitivity and specificity; and (5) Study design (S): Retrospective or prospective studies.

Studies were deemed eligible for exclusion from the meta-analysis if they satisfied the following criteria: (1) Case report, abstract, letter, review, meta-analysis; (2) Irrelevant titles and abstracts; (3) Unable to retrieve data on diagnosis-related metrics, including True-positive (TP), False-positive (FP), False-negative (FN), and True-negative (TN); (4) Non-English publication; (5) Non-suspected recurrence patients; (6) Studies employing other radiotracers (non-FDG).

### 2.3 Quality assessment

To assess the quality of methodologies in the included studies, this meta-analysis followed the Quality Assessment for Studies of Diagnostic Accuracy-2 (QUADAS-2) guidelines, which are specifically designed for evaluating systematic reviews of diagnostic accuracy studies (13), consisting of four critical domains: (1) patient selection; (2) index test; (3) reference standard; and (4) flow and timing. According to QUADAS, the risk of bias in each study was classified as “high risk,” “low risk,” or “unclear risk”.

Two independent authors assessed all included studies, with any discrepancies settled by consulting an additional author to reach an agreement.

### 2.4 Data extraction

The data extracted from the selected studies included as follows: the author, publication year, type of imaging test (PET/CT or PET/MRI), country of the study, study design (prospective or retrospective), analysis type (patient-based or lesion-based), reference standard (pathology and/or follow-up imaging), number of patients, clinical indication stage, mean or median age of patients, previous treatment (such as surgery, chemotherapy, radiotherapy, hormonal therapy, etc.), as well as the diagnostic outcomes including TP, FP, TN, and FN.

Two reviewers independently carried out the data extraction from each study. Disagreements were resolved by discussion, achieving consensus to guarantee precision in the data extraction process.

### 2.5 Outcome measures

In this meta-analysis, the primary outcome measures centered on the diagnostic performance of [^18^F]FDG PET/CT and [^18^F]FDG PET/MRI in detecting breast cancer recurrence. The diagnostic performance of these imaging modalities was evaluated through their sensitivities and specificities. Sensitivity was defined as the proportion of TP imaging results to the total number of TP and FN results. Specificity was defined as the proportion of TN results to the total number of TN and FP results.

### 2.6 Statistical analysis

The present study utilized the recommended standard method for conducting a diagnostic meta-analysis (14). The DerSimonian and Laird method was employed to evaluate specificity and sensitivity, followed by transformation using the Freeman-Tukey double arcsine transformation (15). Confidence intervals were calculated using the Jackson method (16). Heterogeneity within and between groups was assessed with the Cochrane Q and *I*^2^ statistics (17). In cases of significant heterogeneity (*p* < 0.10 or *I*^2^ > 50%), a sensitivity analysis was conducted by sequentially excluding studies to reassess sensitivity or specificity (18).

To evaluate publication bias, funnel plots were used alongside Egger’s test. For heterogeneity tests, *p* < 0.10 was considered significant, while for all other statistical tests, a significance level of *p* < 0.05 was applied. All statistical analyses and graphical representations were performed using R software version 4.4.0.

## 3 Results

### 3.1 Study selection

**Figure 1:**
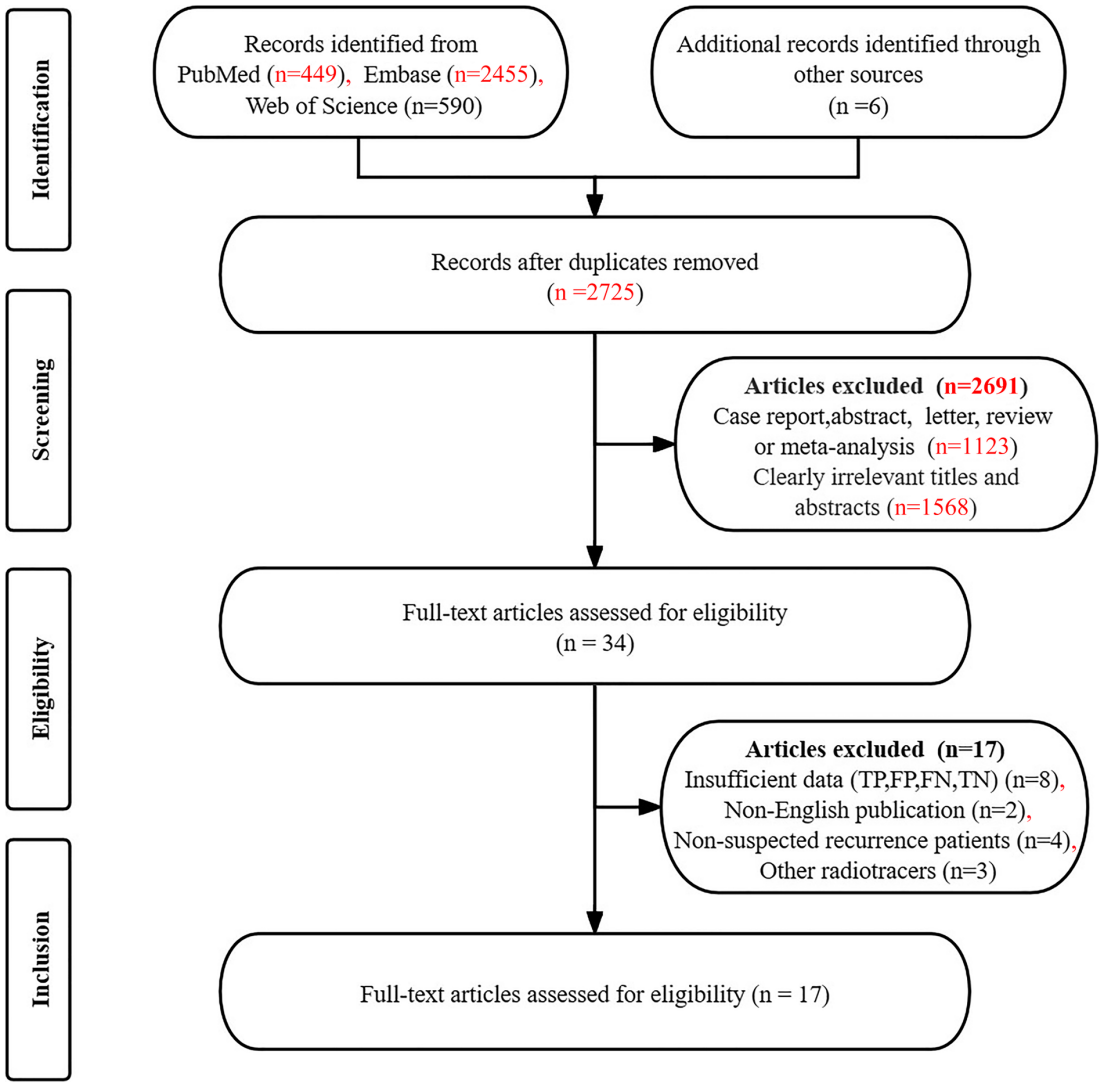
Detailed screening process for included studies.

The initial search yielded 3,500 articles, from which 775 duplicates were excluded. Additionally, 2,725 articles did not meet the established inclusion criteria, leaving 34 articles for further consideration. After a detailed reading of the complete texts of the remaining articles, an additional 17 were excluded for the following reasons: unavailable data on TP, TN, FP, and FN (*n* = 8), non-English publications (*n* = 2), studies involving non-suspected recurrence patients (*n* = 4), and the use of other radiotracers (*n* = 3). Consequently, 17 articles (19–35) were included in the final analysis, which assessed the diagnostic effectiveness of [^18^F]FDG PET/CT (*n* = 16) (19–32, 34, 35) and [^18^F]FDG PET/MRI (*n* = 4) (31–34), including 3 articles (31, 32, 34) that allowed for head-to-head comparison. Figure 1 provides details of the article selection process based on the PRISMA flow diagram.

### 3.2 Study description and quality assessment

This analysis incorporated 17 eligible studies, encompassing a total of 1,450 patients who were being evaluated for suspected recurrence of breast cancer, with ages ranging from 22 to 91.2 years. Of these studies, 11 (65%) were retrospective in nature (19, 21–30), while 6 (35%) were prospective studies (20, 31–35). In terms of analysis methods, 10 studies (59%) performed patient-based analyses (19, 20, 22–24, 26, 27, 30, 31, 33), whereas 7 studies (41%) utilized lesion-based analyses (21, 25, 28, 29, 32, 34, 35). The reference standards applied included pathology and/or imaging follow-up in 16 articles (94%) (19–30, 32–35), with one article (5%) relying solely on imaging follow-up (31). All patients included in these studies were in the post-treatment stage of breast cancer. The previous treatments they had undergone varied and included types such as surgery and chemotherapy. Table 1 summarizes the basic information and patient characteristics of the included studies.

**Table 1:** Study and patient characteristics of the included studies.

Author	Year	Type of imaging test	Patient characteristics							
			Country	Study design	Analysis	Reference standard	No. of patients	Clinical indication	Mean/Median age	Previous treatment
Radan et al.	2006	PET/CT	UK	Retro	PB and LB	Pathology and/or follow-up imaging	46	Post-treatment stage	Mean (range): 59.9 (32–79)	Surgery or Chemotherapy or Radiotherapy or Hormonal therapy
Haug et al.	2007	PET/CT	Germany	Pro	PB	Pathology and/or follow-up imaging	34	Post-treatment stage	Mean (range): 50.5 (28–73)	Surgery
Palomar et al.	2010	PET/CT	Spain	Retro	PB	Pathology and/or follow-up imaging	70	Post-treatment stage	Mean (range): 61.3 (34–84)	Surgery or Hormonal therapy or Follow-up or Chemotherapy
Dirisamer et al.	2010	PET/CT	Austria	Retro	PB	Pathology and/or follow-up imaging	80	Post-treatment stage	Mean (range): 61 (40–84)	Chemotherapy
Champion et al.	2011	PET/CT	France	Retro	PB	Pathology and/or follow-up imaging	228	Post-treatment stage	Mean (range): 60.8 (30–91)	Surgery or Chemotherapy or Endocrine treatment
Filippi et al.	2011	PET/CT	Greece	Retro	PB	Pathology and/or follow-up imaging	46	Post-treatment stage	Mean (range): 57.6 (38–68)	Surgery or Chemotherapy or radio-frequency ablation
Murakami et al.	2012	PET/CT	Japan	Retro	PB	Pathology and/or follow-up imaging	57	Post-treatment stage	Median (range): 50 (35–79)	NA
Manohar et al.	2012	PET/CT	India	Retro	PB	Pathology and/or follow-up imaging	111	Post-treatment stage	Median (range): 52 (22–80)	Surgery
Cervino et al.	2014	PET/CT	Italy	Retro	PB	Pathology and/or follow-up imaging	193	Post-treatment stage	Mean ± SD (Range): 61 ± 12 (32–83)	Surgery and others
Chang et al.	2014	PET/CT	China	Retro	PB	Pathology and/or follow-up imaging	140	Post-treatment stage	Median (range): 51 (33–84)	Surgery or chemotherapy
Cochet et al.	2014	PET/CT	Australia	Retro	PB	Pathology and/or follow-up imaging	63	Post-treatment stage	Mean (range): 57 (29–86)	NA
Dong et al.	2015	PET/CT	China	Retro	PB and LB	Pathology and/or follow-up imaging	26	Post-treatment stage	Mean ± SD (Range): 54.9 ± 12.1 (33–84)	Surgery
Vogsen et al.	2021	PET/CT	Denmark	Pro	PB and LB	Pathology and/or follow-up imaging	225	Post-treatment stage	Median (range): 68(33.3–91.2)	Neoadjuvant and/or adjuvant treatment
Grueneisen et al.	2017	PET/MRI	Germany	Pro	PB and LB	Pathology and/or follow-up imaging	36	Post-treatment stage	Mean ± SD (Range): 58 ± 15 (28–78)	NA
Sawicki et al.	2016	PET/CT vs. PET/MRI	Germany	Pro	PB and LB	Pathology and/or follow-up imaging	21	Post-treatment stage	Mean ± SD (Range): 54.9 ± 11.5 (38.5–76.9)	NA
Melsaether et al.	2016	PET/CT vs. PET/MRI	US	Pro	PB	Follow-up imaging	51	Post-treatment stage	Mean (range): 56(32–76)	NA
Rezk et al.	2019	PET/CT vs. PET/MRI	Egypt	Pro	LB	Pathology and/or follow-up imaging	23	Post-treatment stage	Mean (range): 56(47–65)	NA

PB, patient-based; LB, lesion-based; Pro, prospective; Retro, retrospective; NA, not available.

**Figure 2:**
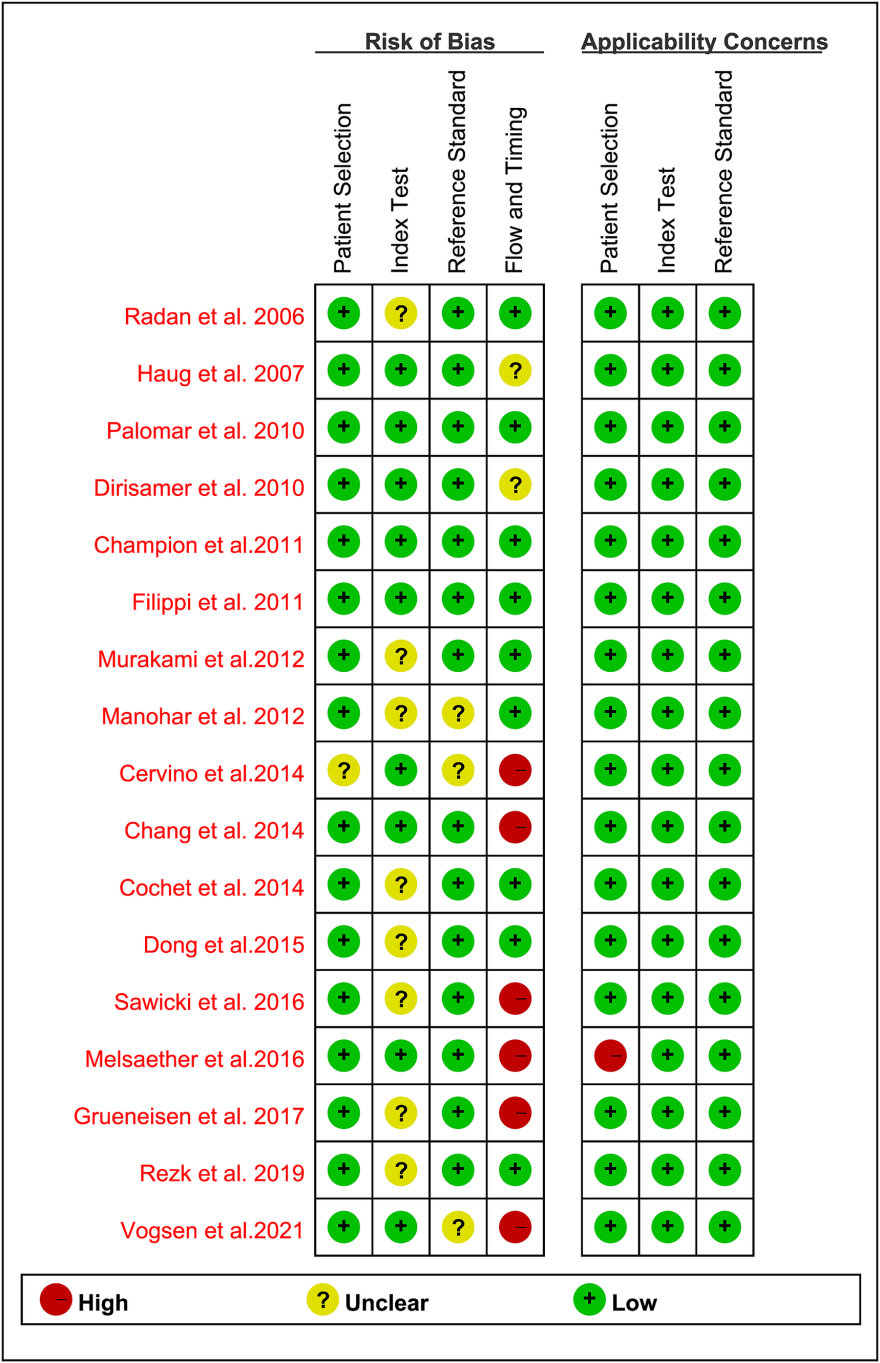
Summary of risk of bias and applicability concerns of all included studies according to QUADAS-2 tool.

Based on the QUADAS-2 tool, the risk of bias in all studies is illustrated in Figure 2. In assessing the risk of bias related to patient selection, index testing, and reference standards, the majority of the articles were deemed low risk, with no articles classified as high risk. Regarding the flow and timing aspect, 6 studies (35%) (27, 28, 31–33, 35) were judged to be at “high risk” due to a time interval exceeding 3 months. Overall, no significant quality issues were identified in the included studies.

### 3.3 Lesion-based comparison of the sensitivity of [^18^F]FDG PET/CT and [^18^F]FDG PET/MRI in breast cancer recurrence

This meta-analysis assesses the diagnostic efficacy of [^18^F]FDG PET/CT and [^18^F]FDG PET/MRI in detecting recurrence of breast cancer at the lesion level. The analysis incorporates data from six studies (19, 30, 32–35), encompassing a total of 377 patients and 1,313 lesions. Specifically, five studies (19, 30, 32, 34, 35) involving 341 patients and 829 lesions employed [^18^F]FDG PET/CT, whereas three studies (32–34) with 80 patients and 484 lesions utilized [^18^F]FDG PET/MRI.

**Figure 3:**
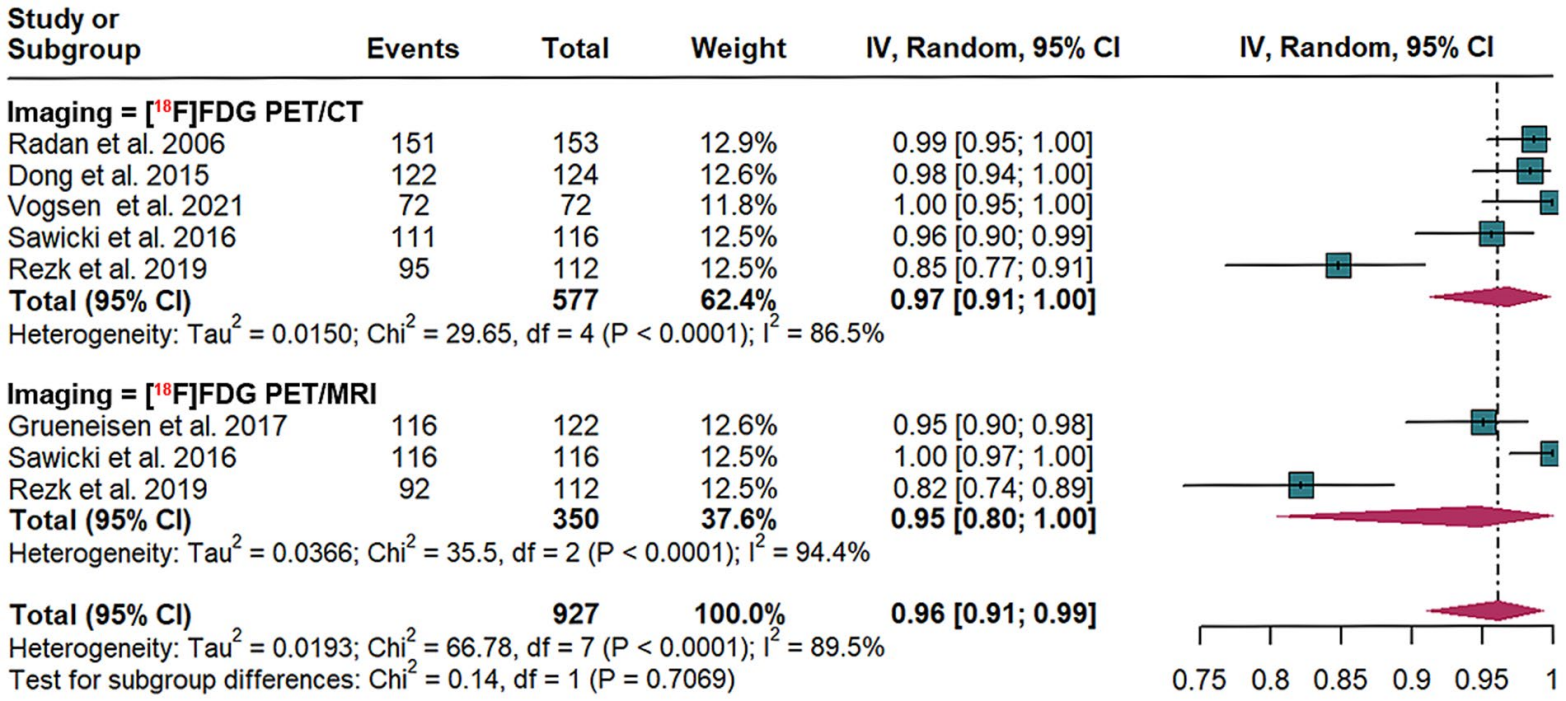
Lesion-based forest plot illustrating the sensitivity of [^18^F]FDG PET/CT and [^18^F]FDG PET/MRI for detecting breast cancer recurrence.

The pooled sensitivity for [^18^F]FDG PET/CT was 0.97 (95% CI: 0.91–1.00). In comparison, [^18^F]FDG PET/MRI demonstrated a pooled sensitivity of 0.95 (95% CI: 0.91–0.99). The comparative analysis of pooled sensitivity between [^18^F]FDG PET/CT and [^18^F]FDG PET/MRI revealed no statistically significant difference (*p* = 0.71) (Figure 3).

For lesion-based [^18^F]FDG PET/CT, heterogeneity had an *I*^2^ of 86.5%, with stable results in the leave-one-out sensitivity analysis (Supplementary Figure S1).

### 3.4 Lesion-based comparing the specificity of [^18^F]FDG PET/CT and [^18^F]FDG PET/MRI in breast cancer recurrence

**Figure 4:**
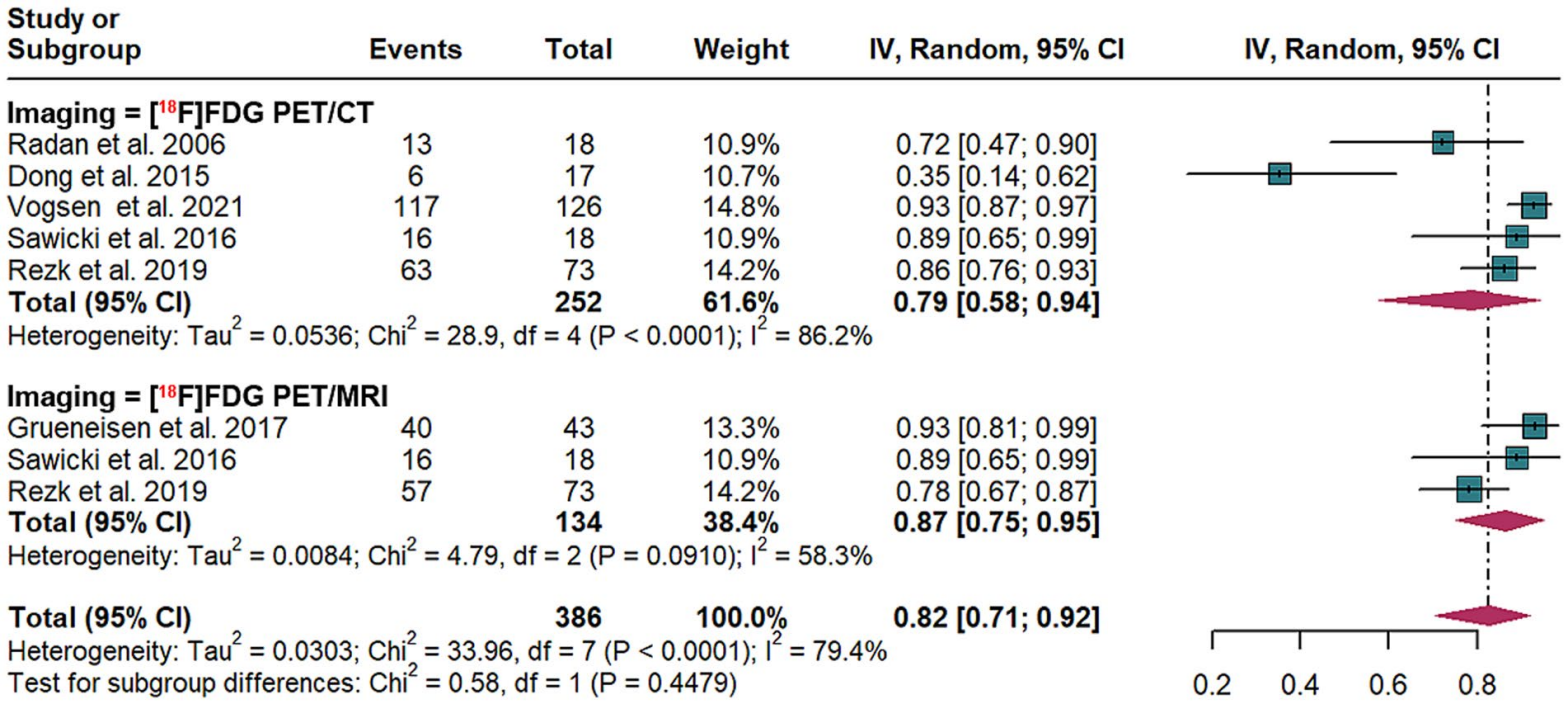
Lesion-based forest plot illustrating the specificity of [^18^F]FDG PET/CT and [^18^F]FDG PET/MRI for detecting breast cancer recurrence.

In the detection of recurrence for breast cancer, at the lesion level, the overall specificity of [^18^F]FDG PET/CT was 0.79 (95% CI: 0.58–0.94), compared to a pooled specificity of 0.87 (95% CI:0.75–0.95) for [^18^F]FDG PET/MRI. The total specificity of [^18^F]FDG PET/CT and [^18^F]FDG PET/MRI showed no statistical difference (*p* = 0.44) (Figure 4).

For [^18^F]FDG PET/CT, the lesion-based specificity exhibited *I*^2^ values of 70%. The leave-one-out sensitivity analysis revealed that the results remained stable (Supplementary Figure S2).

### 3.5 Patient-based comparison of the sensitivity of [^18^F]FDG PET/CT and [^18^F]FDG PET/MRI in breast cancer recurrence

This meta-analysis evaluates the diagnostic efficacy of [^18^F]FDG PET/CT and [^18^F]FDG PET/MRI for the detection of breast cancer recurrence at the patient level. The analysis synthesizes data from 16 studies (19–33, 35), which collectively included 1,427 patients. Specifically, 15 studies (19–32, 35) involving 1,391 patients utilized [^18^F]FDG PET/CT, while three studies (31–33) with 108 patients employed [^18^F]FDG PET/MRI.

**Figure 5:**
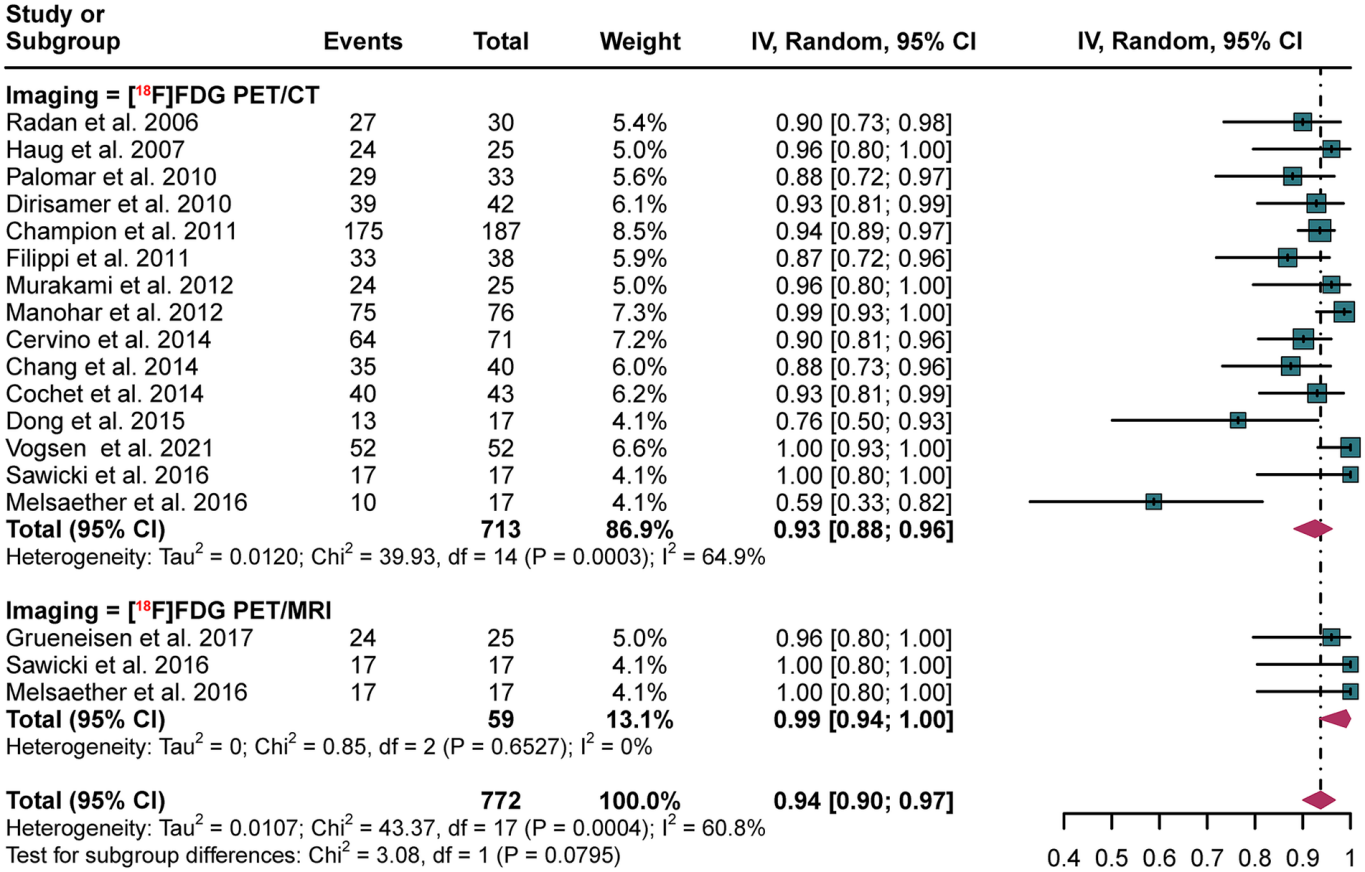
Patient-based forest plot illustrating the sensitivity of [^18^F]FDG PET/CT and [^18^F]FDG PET/MRI for detecting breast cancer recurrence.

In diagnosing recurrence in breast cancer at the patient level, [^18^F]FDG PET/CT demonstrated a pooled sensitivity of 0.93 (95% CI: 0.88–0.96), while [^18^F]FDG PET/MRI showed an overall sensitivity of 0.99 (95% CI: 0.94–1.00). Statistical analysis revealed no significant difference in the overall sensitivity between [^18^F]FDG PET/CT and [^18^F]FDG PET/MRI (*p* = 0.07) (Figure 5).

In patient-based [^18^F]FDG PET/CT analyses, heterogeneity was quantified with an *I*^2^ value of 64.9%, and the results remained consistent upon conducting a leave-one-out sensitivity analysis (Supplementary Figure S3).

### 3.6 Patient-based comparison of the specificity of [^18^F]FDG PET/CT and [18F]FDG PET/MRI in breast cancer recurrence

**Figure 6:**
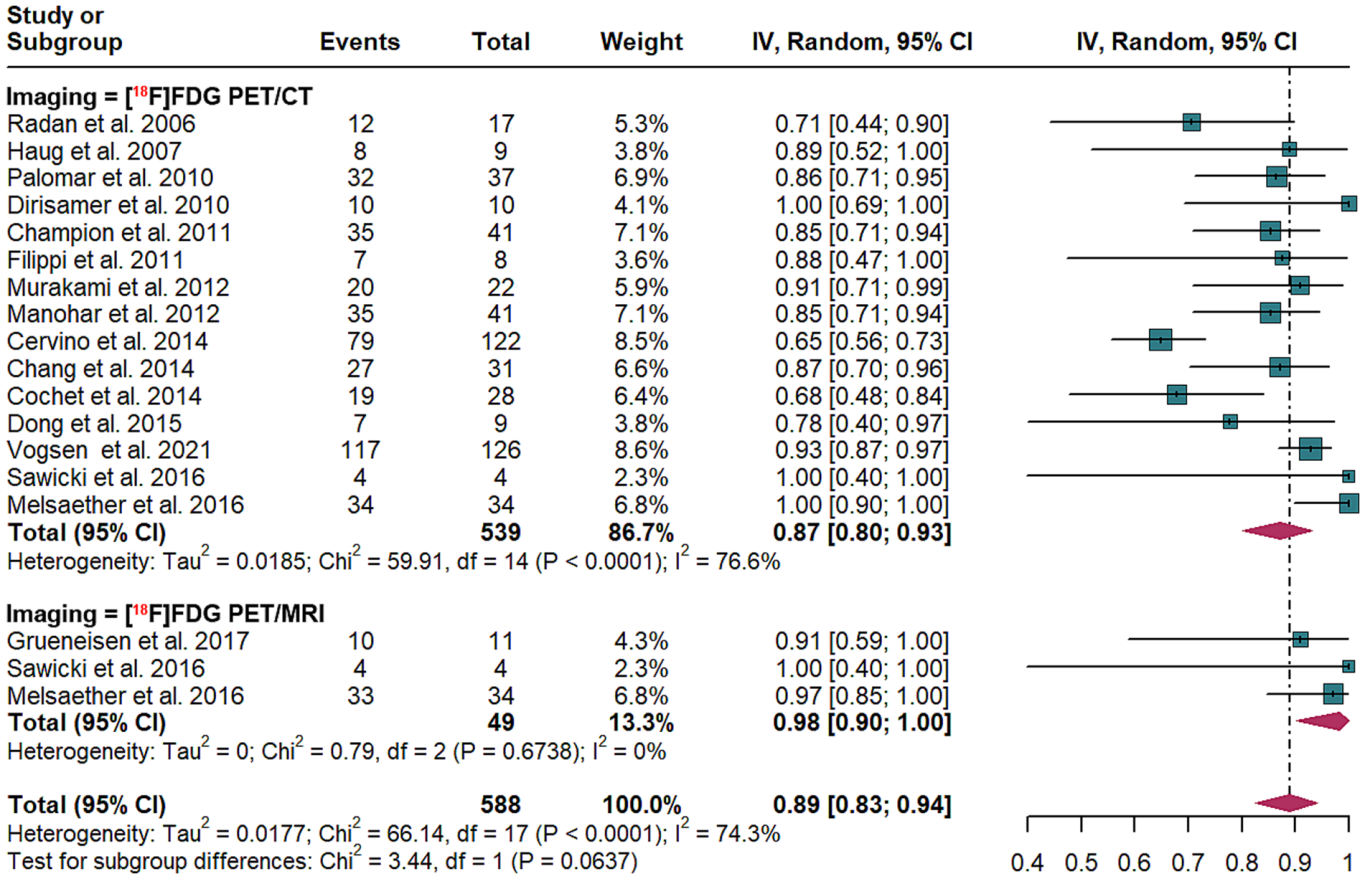
Patient-based forest plot illustrating the head-to-head comparison of the specificity of [^18^F]FDG PET/CT and [^18^F]FDG PET/MRI for detecting breast cancer recurrence.

In the context of diagnosing breast cancer recurrence at the patient level, the aggregated specificity of [^18^F]FDG PET/CT was determined to be 0.87 (95% CI: 0.80–0.93). In comparison, [^18^F]FDG PET/MRI demonstrated an overall specificity of 0.98 (95% CI: 0.90–1.00). Statistical analysis indicated no significant difference in specificity between the two imaging modalities (*p* = 0.06) (Figure 6).

In analyses of patient-based [^18^F]FDG PET/CT, heterogeneity was quantified with an *I*^2^ value of 76.6%. The results demonstrated robustness, as evidenced by their consistency in a leave-one-out sensitivity analysis (Supplementary Figure S4).

### 3.7 Publication bias

The funnel plot and Egger’s test demonstrated an absence of evidence for publication bias regarding the sensitivity and specificity of [^18^F]FDG PET/CT on both lesion-based and patient-based analyses, respectively (all *p* > 0.50) (Supplementary Figures S5–S8).

## 4 Discussion

Current guidelines from prominent oncology organizations, including the American Society of Clinical Oncology, the National Comprehensive Cancer Network, and the European Society for Medical Oncology, advise that follow-up care for breast cancer patients who are asymptomatic should primarily consist of regular physical examinations and annual mammographic screenings. The use of additional laboratory or imaging tests on a routine basis is not recommended within these guidelines (36–38). However, for breast cancer patients with a suspected recurrence, there is currently no standardized follow-up protocol in place, making additional radiologic imaging crucial, particularly when recurrence is suggested by elevated tumor markers or concerning symptoms.

A prospective study evaluated [^18^F]FDG PET/CT in 100 patients with suspected breast cancer recurrence compared to thoracoabdominal contrast-enhanced CTs (ceCT) and bone scans (39). The findings revealed that 22% of patients were diagnosed with distant recurrence, 19% had local recurrence only, and 59% exhibited no evidence of recurrence. The diagnostic accuracy for identifying distant recurrence was assessed using the area under the ROC curve, with [^18^F]FDG PET/CT achieving an AUC of 0.99, compared to 0.84 for ceCT and 0.86 for the combination of ceCT and bone scans (39). Another study reported that [^18^F]FDG PET/CT exhibits a high positive predictive value of 0.97 and an accuracy ranging from 0.83 to 0.86 in detecting suspected recurrent condition (24, 40). Additionally, other clinical guidelines suggest that [^18^F]FDG PET/CT can be particularly useful in identifying the site of recurrence when conventional imaging methods yield inconclusive results (41). In contrast, another meta-analysis has demonstrated that [^18^F]FDG PET/MRI offers robust diagnostic accuracy, with a sensitivity of 0.94 and specificity of 0.90 for nodal staging, as well as a sensitivity of 0.98 and specificity of 0.96 for distant staging (42). Preliminary data suggest that PET/MRI could be a viable alternative to PET/CT for current clinical applications (43). In conclusion, both [^18^F]FDG PET/CT and [^18^F]FDG PET/MRI perform well in detecting breast cancer recurrence. However, a systematic comparison is still lacking, leaving the question of which modality is superior unresolved. This meta-analysis seeks to fill a gap in the literature by comparing the diagnostic sensitivity and specificity of [^18^F]FDG PET/CT and [^18^F]FDG PET/MRI in breast cancer recurrence.

The meta-analytic outcomes of this systematic review demonstrate that there is no statistically significant difference in overall sensitivity between [^18^F]FDG PET/CT and [^18^F]FDG PET/MRI for the detection of breast cancer recurrence at both the lesion level (*p* = 0.71) and the patient level (*p* = 0.07). Similarly, the overall specificity of the two imaging modalities does not differ significantly at the lesion level (*p* = 0.44) or the patient level (*p* = 0.06). These findings indicate that both imaging techniques provide comparable diagnostic performance in clinical settings. These findings collectively indicate that both imaging techniques demonstrate equivalent diagnostic accuracy in the detection of recurrent breast cancer within clinical practice.

The diagnostic equivalency observed can be attributed to the common use of [^18^F]FDG, a glucose analog that preferentially accumulates in metabolically active tumor cells (44). While PET/MRI provides enhanced soft tissue contrast, especially in breast and pelvic areas, the diagnostic efficacy is predominantly determined by the metabolic imaging aspect (45, 46). Consequently, the PET-based assessment of glucose metabolism is likely the primary determinant of sensitivity and specificity in both imaging modalities.

In addition, at the lesion-based analysis, a study assessing the performance of these imaging modalities revealed that [^18^F]FDG PET/MRI correctly identified a greater proportion of lesions, achieving a sensitivity of 98.5% compared to 94.8% for [^18^F]FDG PET/CT (32). This finding suggests that PET/MRI may be more effective in detecting smaller or less conspicuous lesions that could be overlooked by PET/CT (32). Furthermore, patient-based analyses indicate that [^18^F]FDG PET/MRI offers advantages over PET/CT. A meta-analysis comparing these modalities found that [^18^F]FDG PET/MRI exhibited higher sensitivity for detecting distant metastases in breast cancer patients, with a sensitivity of 1.00 compared to 0.96 for PET/CT (47).

To date, no comprehensive meta-analysis or extensive comparative study has specifically investigated the influence of recurrence site on the relative diagnostic efficacy of [^18^F]FDG PET/CT versus PET/MRI. This omission constitutes a significant gap in the existing literature, as elucidating lesion-specific diagnostic advantages could facilitate the development of more personalized and anatomically targeted imaging strategies for patients with suspected recurrence. In response to this gap, the current study is pioneering in its examination of the impact of recurrence site on imaging modality performance through stratified subgroup analyses, providing novel insights into how anatomical context may affect diagnostic sensitivity and specificity. In our meta-analysis, both analytical methodologies produced consistent results, demonstrating no statistically significant difference in sensitivity or specificity between [^18^F]FDG PET/CT and PET/MRI at either the lesion level or the patient level. This internal consistency enhances confidence in the robustness of our findings and indicates that the diagnostic equivalence between these modalities is maintained irrespective of the level of analytical granularity. However, future research should clearly differentiate between these analytical levels and, where feasible, employ hierarchical modeling approaches that account for the clustering of lesions within patients.

Furthermore, this meta-analysis offers a thorough assessment of the diagnostic effectiveness of [^18^F]FDG PET/CT and [^18^F]FDG PET/MRI for detecting breast cancer recurrence, filling critical gaps from earlier research. Xiao et al. (48) conducted a systematic review and meta-analysis, demonstrating the high sensitivity (0.90) and specificity (0.81) of [^18^F]FDG PET/CT in detecting breast cancer recurrence. While their findings highlighted the diagnostic value of [^18^F]FDG PET/CT, they did not compare it with PET/MRI, leaving unresolved the question of which modality performs better in this context. Our study builds upon their work by providing the first head-to-head comparison between PET/CT and PET/MRI, offering a clearer, evidence-based understanding of the diagnostic capabilities of both modalities in breast cancer recurrence.

In contrast, Dan et al. (49) focused on the diagnostic performance of PET/MRI in breast cancer, including initial diagnosis, lymph node involvement, and bone metastasis. While their study demonstrated superior sensitivity (0.95) and specificity (0.94) of PET/MRI for breast lesions and metastases, a direct comparison between PET/MRI and PET/CT was not conducted, leaving an important gap in understanding their relative performance.

In comparing the diagnostic efficacy of [^18^F]FDG PET/CT and [^18^F]FDG PET/MRI in breast cancer recurrence, both modalities demonstrated similar sensitivity and specificity, indicating comparable performance in detecting recurrent disease. Nonetheless, each option has its unique benefits and drawbacks. Several studies have demonstrated that PET/CT is a cost-effective and widely available option, including one emphasizing its utility for monitoring metastatic breast cancer treatment responses (50). PET/CT also offers quicker scan times, reducing patient discomfort and rendering it a viable option in numerous clinical practice. A PET/MRI scan, on the other hand, enhances the contrast of soft tissue while reducing radiation exposure, which is especially important for younger or more vulnerable patients (51). In light of these factors, while PET/CT appears to be more economical and accessible, PET/MRI’s enhanced diagnostic performance and safety profile in specific cases cannot be ignored. The complementary strengths of these modalities suggest that combining them into a hybrid diagnostic approach may improve overall diagnostic accuracy. Patients’ clinical circumstances and diagnostic needs should ultimately determine whether PET/CT or PET/MRI is appropriate.

Interpreting the results of this meta-analysis requires consideration of certain limitations. First, the heterogeneity among the included studies may have influenced the pooled sensitivities and specificities of [^18^F]FDG PET/CT and [^18^F]FDG PET/MRI. In order to identify potential sources of heterogeneity and evaluate the robustness of our findings, we conducted a leave-one-out sensitivity analysis. The results demonstrated stability across all iterations, with no individual study exerting a disproportionate influence on the aggregated sensitivity or specificity estimates. This consistency indicates that our meta-analytic conclusions are robust and not significantly impacted by any outlier study. Moreover, the imbalance in studies between [^18^F]FDG PET/CT (16 studies) and [^18^F]FDG PET/MRI (4 studies) may introduce bias, with PET/CT’s larger sample size potentially affecting statistical power and confidence intervals for PET/MRI. We conducted subgroup analyses by anatomical regions and sample sizes to address this, but the disparity still limits our analysis’s robustness. Future research should aim for more balanced and larger sample sizes for both imaging methods to ensure more reliable results. Another key limitation is the reliance on retrospective studies (9 out of 16), which are susceptible to selection and verification biases, potentially impacting our findings. Although prospective cohort studies would be preferable, the unpredictable nature of breast cancer recurrence makes them impractical. Thus, we used retrospective data despite its limitations. Recognizing these biases is crucial, and future research should prioritize prospective, multi-center cohort studies to minimize biases and improve result generalizability. The absence of standardized imaging protocols across studies is a limitation, as variations in MRI sequences and PET methods may lead to inconsistent diagnostic performance. A universally accepted protocol for imaging breast cancer recurrence is lacking. Developing such standardized protocols should be a research priority to enhance result consistency and enable reliable comparisons between imaging modalities. Lastly, while we assessed publication bias using a funnel plot and Egger’s test, it’s crucial to recognize that studies favoring PET/MRI may dominate the literature, potentially inflating its perceived diagnostic efficacy. Research on breast cancer recurrence often emphasizes imaging techniques like PET/MRI, leading to a higher likelihood of publishing positive results. This bias can misrepresent PET/MRI’s true effectiveness, as studies with negative or inconclusive outcomes may be underreported. Future research should aim to include both positive and negative findings to provide a more accurate evaluation of PET/MRI’s clinical value in detecting breast cancer recurrence.

To overcome the discussed limitations, we suggest larger, balanced studies, standardized imaging protocols, detailed site comparisons, and reducing publication bias. These measures are crucial for enhancing the diagnostic accuracy of [^18^F]FDG PET/CT and [^18^F]FDG PET/MRI in detecting breast cancer recurrence, thereby improving oncology clinical decisions.

## 5 Conclusion

The findings of our meta-analysis suggest that [^18^F]FDG PET/CT and [^18^F]FDG PET/MRI exhibit comparable sensitivity and specificity in diagnosing breast cancer recurrence at both the lesion and patient levels. In a clinical setting, both of these imaging techniques have their respective strengths and limitations, and physicians should take these into account when making the most suitable choice for patients. Considering that the number of articles on [^18^F]FDG PET/MRI in diagnosing recurrence of breast cancer is fewer compared to [^18^F]FDG PET/CT, more head-to-head researches are needed to expand this field to obtain more robust and comprehensive results.

## Data Availability

The raw data supporting the conclusions of this article will be made available by the authors, without undue reservation.
