# Supplementary material for: Comparing the diagnostic efficacy of [18F]FDG PET/CT and [18F]FDG PET/MRI in breast cancer recurrence: a systematic review and meta-analysis
Source: Front Med (Lausanne). 2025 Aug 1;12:1602415. doi: 10.3389/fmed.2025.1602415 (PMC12354408; doi:10.3389/fmed.2025.1602415)
Supplement: Supplementary file 1 [file Data_Sheet_1.docx]

Supplementary Table 1 Search strategy in PubMed, Embase, and Web of Science.

| Database | Search strategy |
| --- | --- |
| PubMed | ("Breast Neoplasms"[Mesh] OR “Breast Neoplasm”[Title/Abstract] OR “Breast Tumor”[Title/Abstract] OR “Breast Cancer” [Title/Abstract] OR “Breast Carcinoma” [Title/Abstract] OR “Mammary Cancer”[Title/Abstract] OR “Mammary Carcinoma” [Title/Abstract] OR “Mammary Neoplasm”[Title/Abstract]) AND ("Positron-Emission Tomography"[Mesh] OR “PET”[Title/Abstract] OR “positron emission tomography”[Title/Abstract]) AND ("Recurrence"[Mesh] OR “recurrent”[Title/Abstract] OR “relapse”[Title/Abstract] OR “Recrudescence”[Title/Abstract]) |
| Embase | (‘breast tumor’/exp OR ‘Breast Neoplasm’:ab,ti OR ‘Breast Tumor’:ab,ti OR ‘Breast Cancer’:ab,ti OR ‘Breast Carcinoma’:ab,ti OR ‘Mammary Cancer’:ab,ti OR ‘Mammary Carcinoma’:ab,ti OR ‘Mammary Neoplasm’:ab,ti) AND ('positron emission tomography'/exp OR ‘positron emission tomography’:ab,ti OR ‘PET’:ab,ti) AND ('cancer recurrence'/exp OR ‘recurrent’:ab,ti OR ‘relapse’:ab,ti OR ‘recrudescence’:ab,ti) |
| Web of Science | ((AB=(“Breast Neoplasms” OR “Breast Neoplasm” OR “Breast Tumor” OR “Breast Cancer” OR “Breast Carcinoma” OR “Mammary Cancer” OR “Mammary Carcinoma” OR “Mammary Neoplasm”)) AND AB=(“positron emission tomography” OR “PET”)) AND AB=(“Recurrence” OR “recurrent” OR “relapse” OR “Recrudescence”) |

Supplementary Figure 1 The leave-one-out lesion-based sensitivity analysis plot of [^18^F]PET/CT sensitivity using the leave-one-out method.


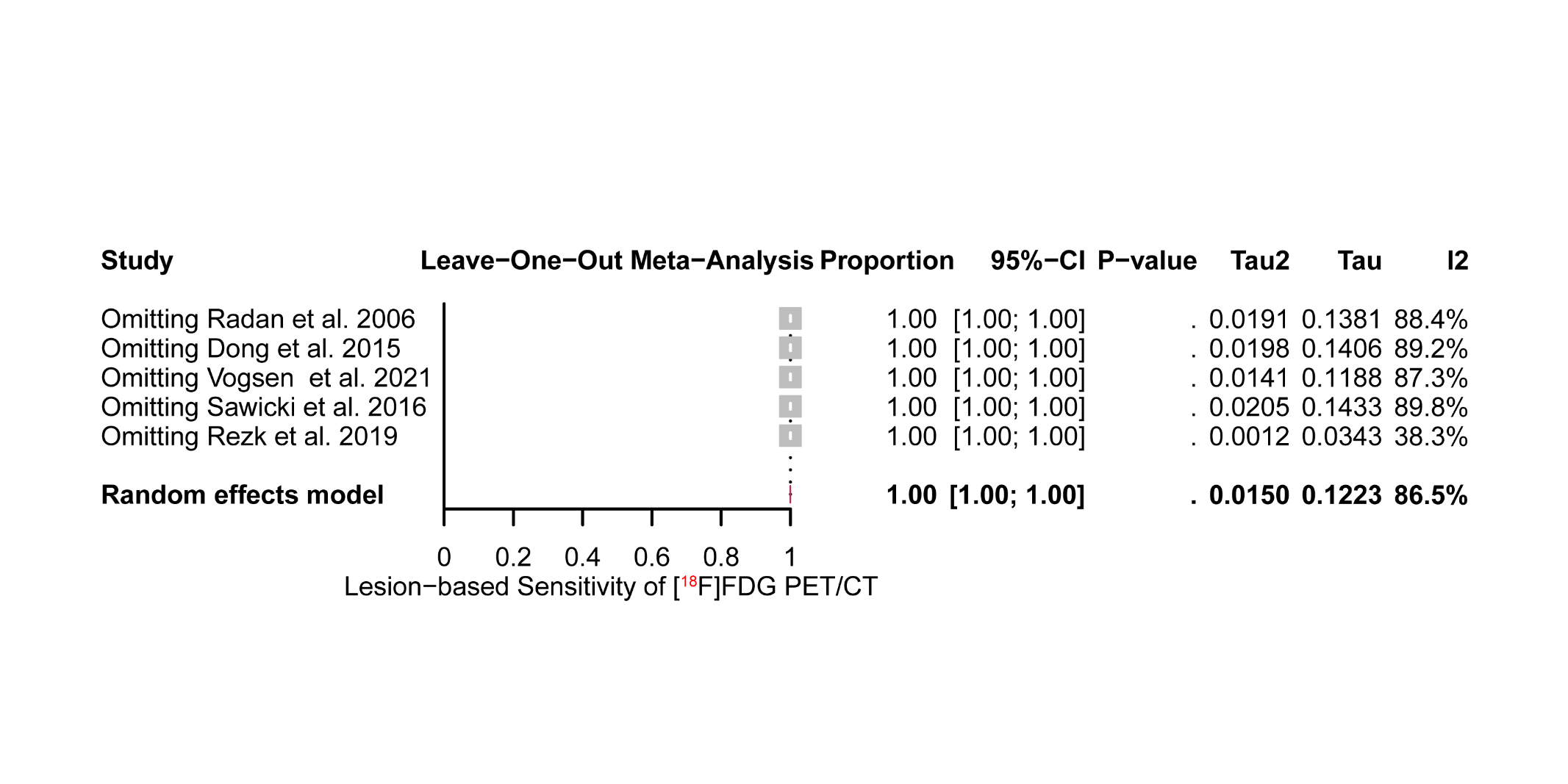


Supplementary Figure 2 The leave-one-out lesion-based sensitivity analysis plot of [^18^F]PET/CT specificity using the leave-one-out method.


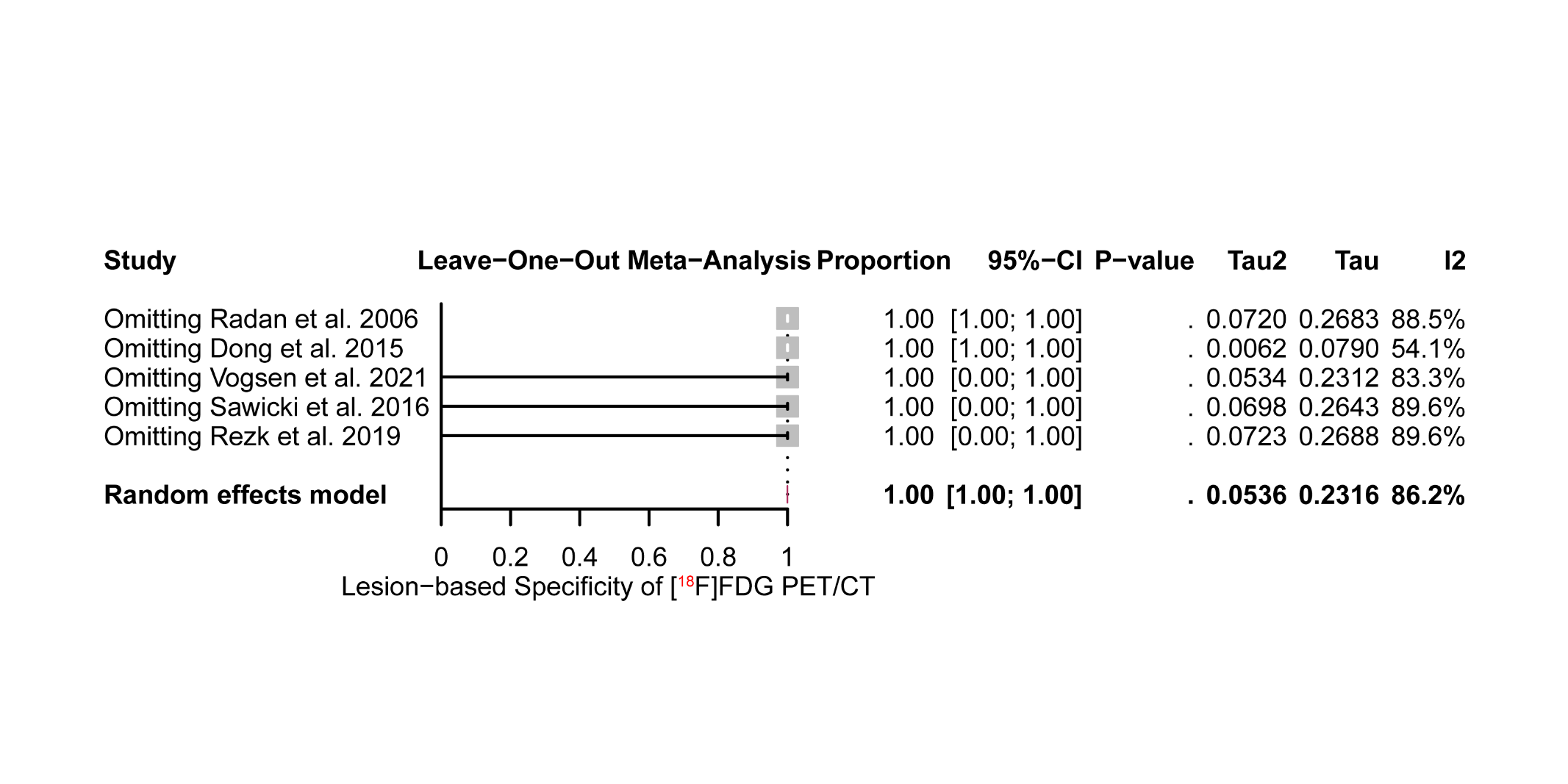


Supplementary Figure 3 The leave-one-out patient-based sensitivity analysis plot of [^18^F]PET/CT sensitivity using the leave-one-out method.


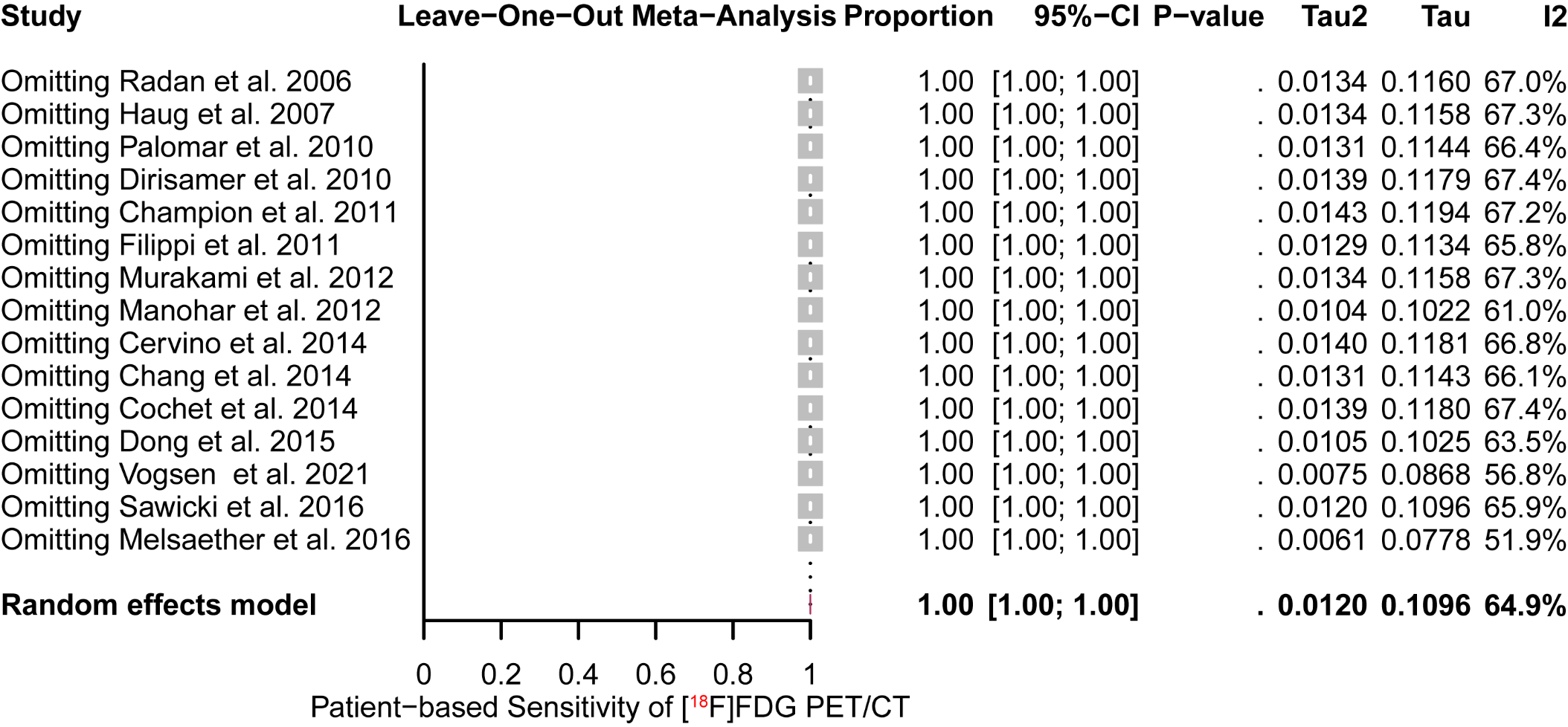


Supplementary Figure 4 The leave-one-out patient-based sensitivity analysis plot of [^18^F]PET/CT specificity using the leave-one-out method.


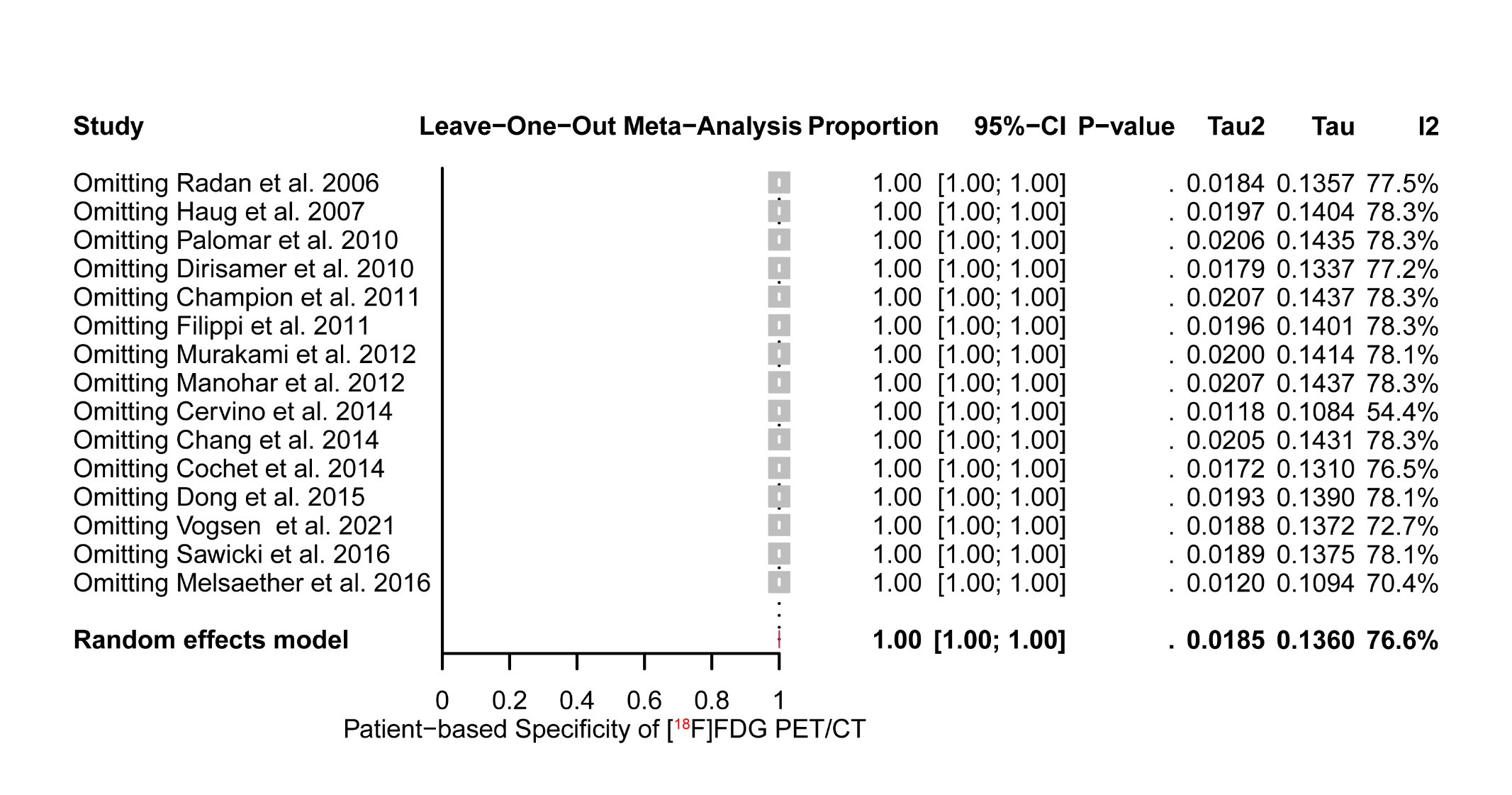


Supplementary Figure 5 Funnel plot of [^18^F]PET/CT sensitivity at the lesion-based level.


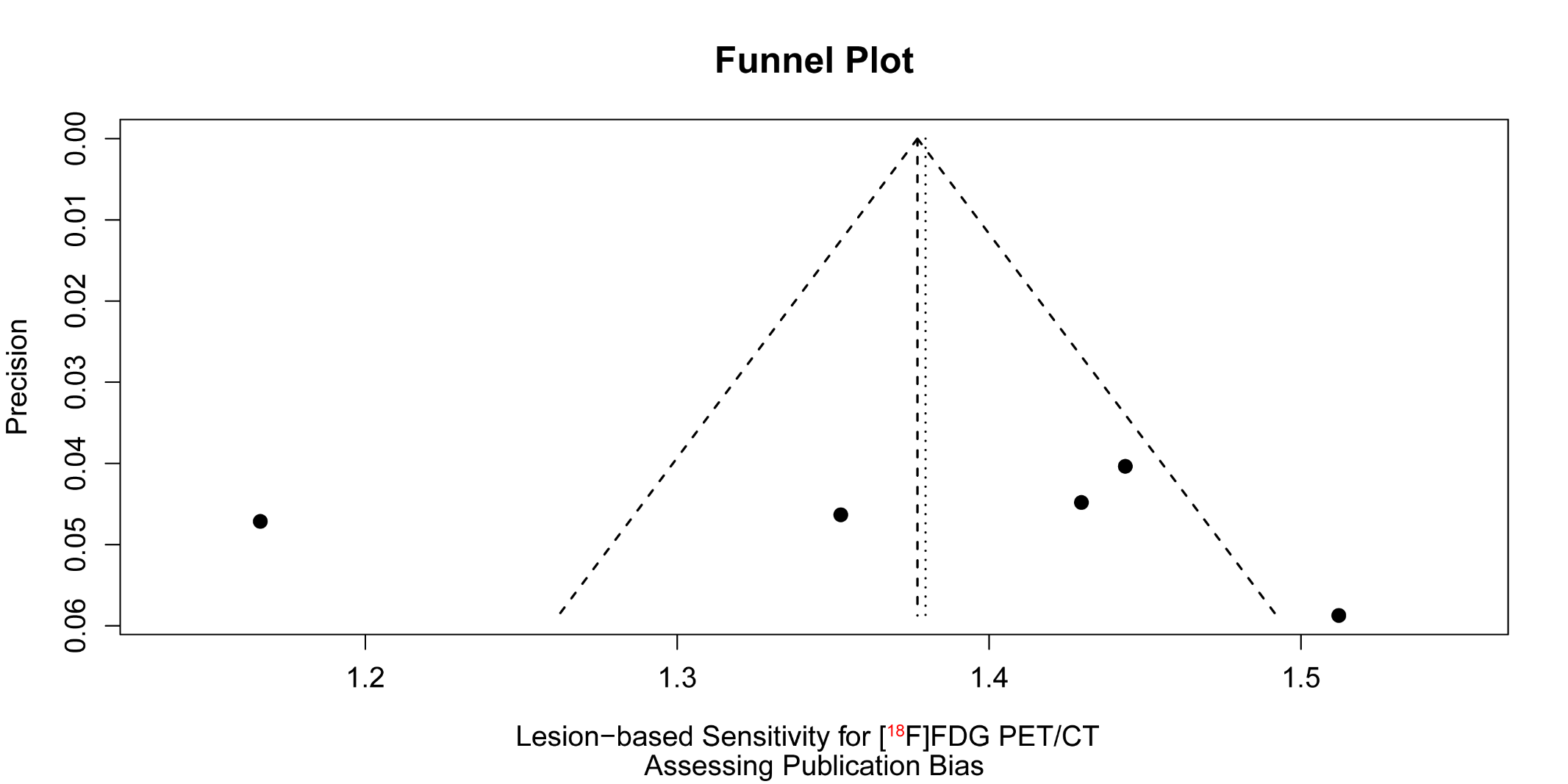


Supplementary Figure 6 Funnel plot of [^18^F]PET/CT specificity at the lesion-based level.


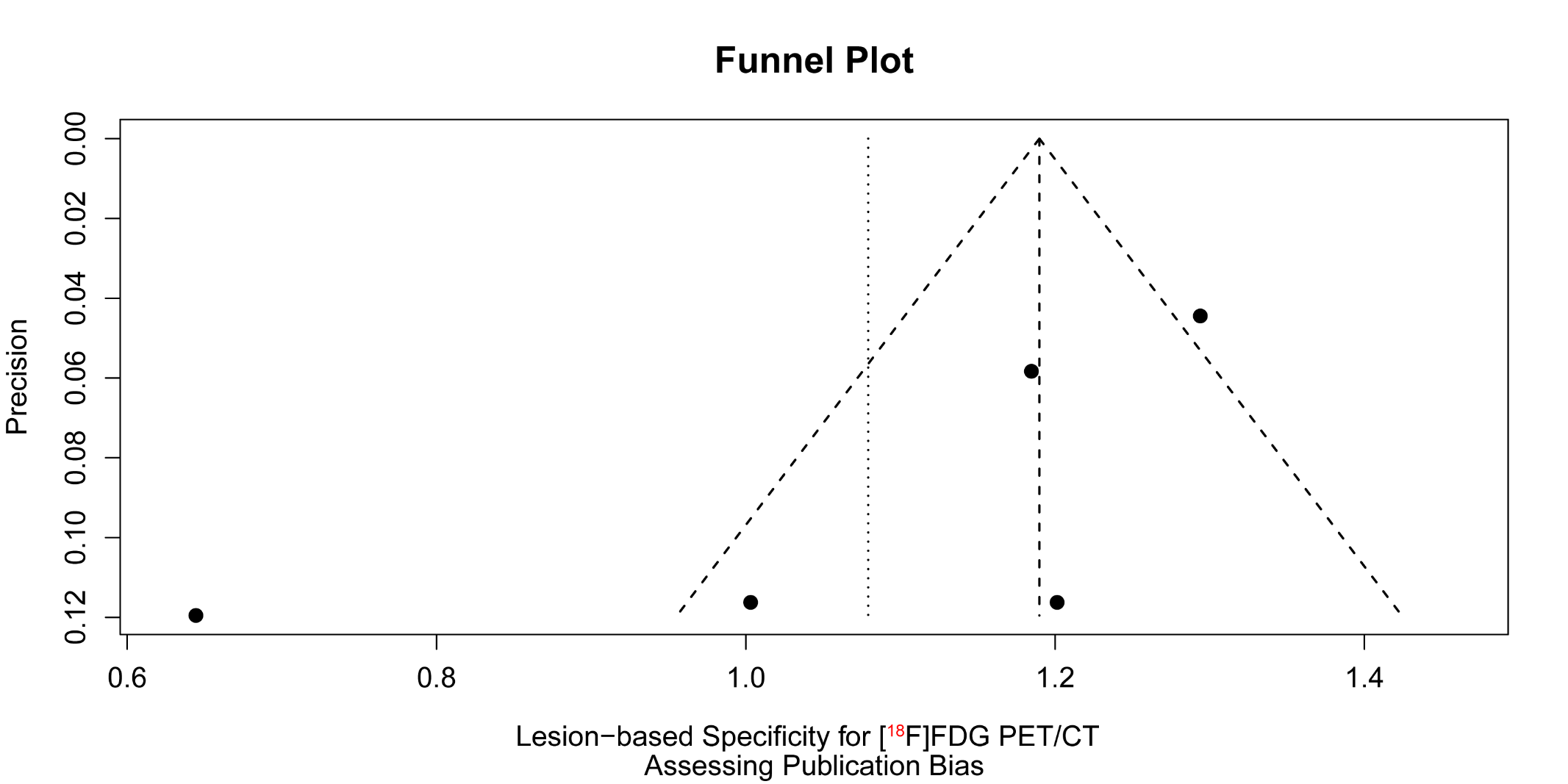


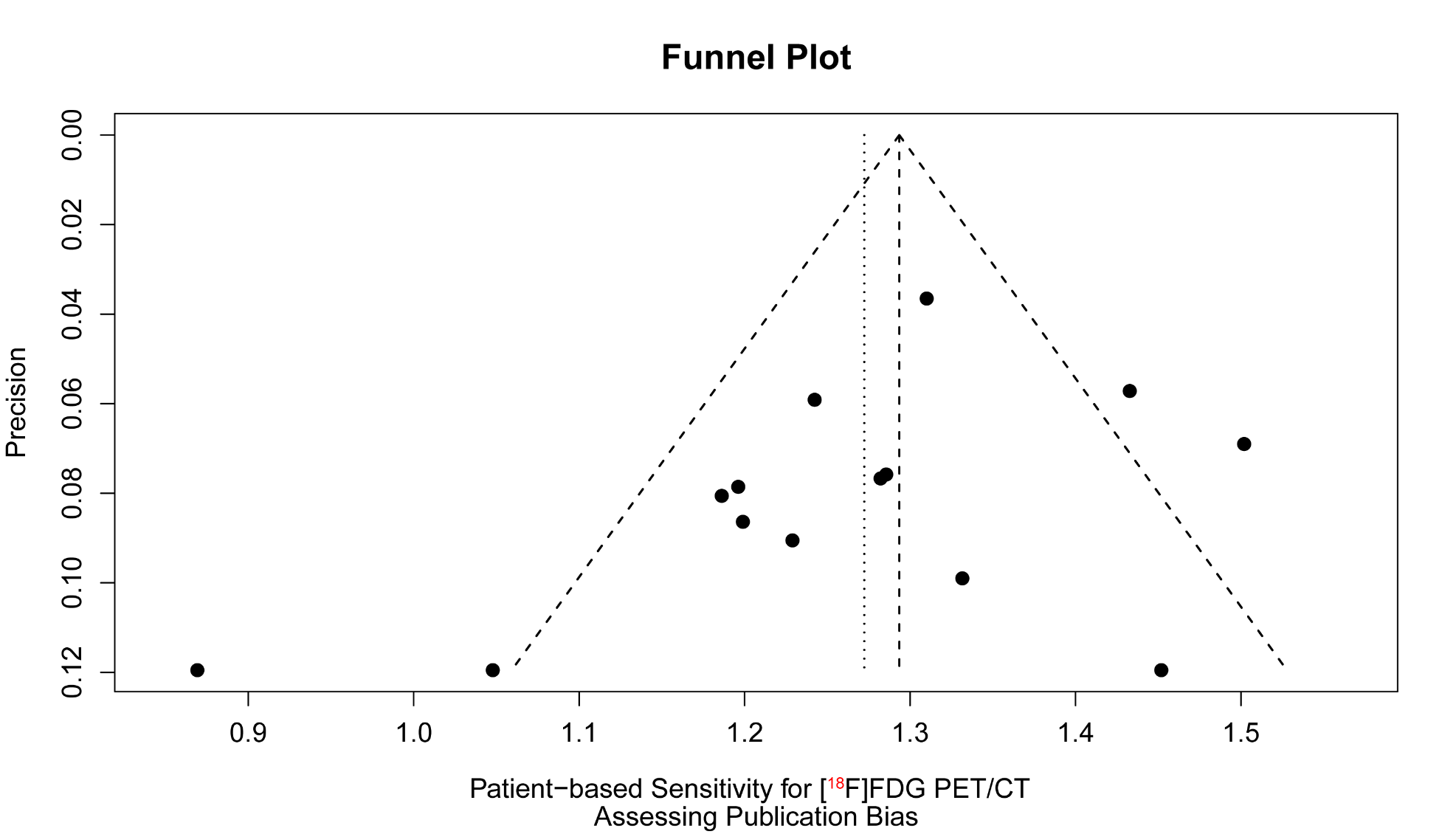
Supplementary Figure 7 Funnel plot of [^18^F]PET/MRI sensitivity at the patient-based level.


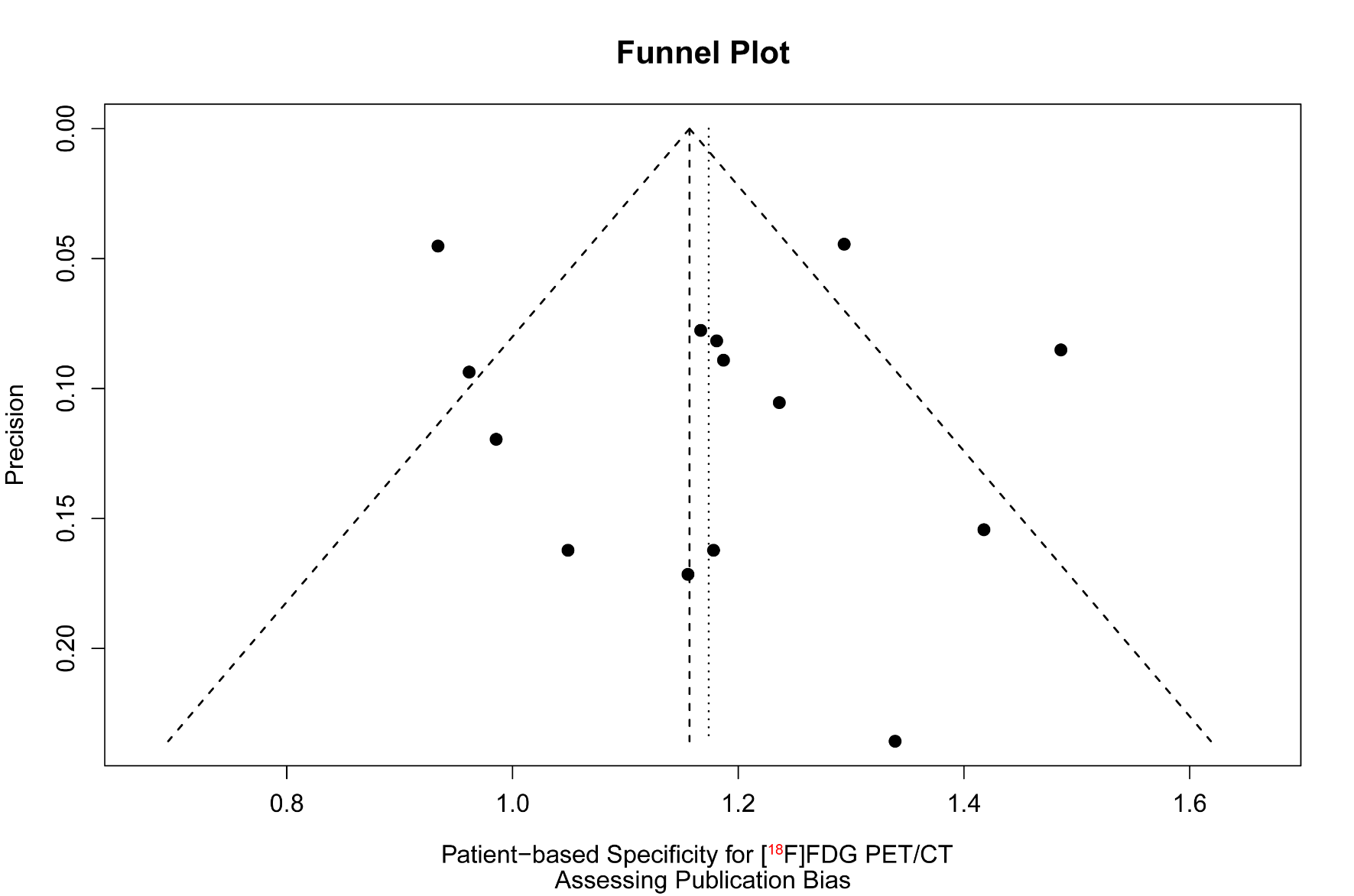
Supplementary Figure 8 Funnel plot of [^18^F]PET/MRI specificity at the patient-based level.
